# Impact of Economic Conditions and Crises on Mortality and its Predictability

**DOI:** 10.1007/s11577-015-0323-8

**Published:** 2015-09-21

**Authors:** Christina Bohk, Roland Rau

**Affiliations:** University of Rostock, Ulmenstrasse 69, 18057 Rostock, Germany

**Keywords:** Economic conditions, Crisis, Life expectancy, Projections, Spain, Hungary, Russia, Ökonomische Bedingungen, Krisen, Lebenserwartung, Prognosen, Spanien, Ungarn, Russland

## Abstract

To investigate how economic conditions and crises affect mortality and its predictability in industrialized countries, we review the related literature, and we forecast mortality developments in Spain, Hungary, and Russia—three countries which have recently undergone major transformation processes following the introduction of radical economic and political reforms. The results of our retrospective mortality forecasts from 1991 to 2009 suggest that our model can capture major changes in long-term mortality trends, and that the forecast errors it generates are usually smaller than those of other well-accepted models, like the Lee-Carter model and its coherent variant. This is because our approach is capable of modeling (1) dynamic shifts in survival improvements from younger to older ages over time, as well as (2) substantial changes in long-term trends by optionally complementing the extrapolated mortality trends in a country of interest with those of selected reference countries. However, the forecasting performance of our model is limited (like that of every model): e.g., if mortality becomes extremely volatile—as was the case in Russia after the dissolution of the Soviet Union—generating a precise forecast will depend more on luck than on methodology and expert judgment. In general, we conclude that, on their own, recent economic changes appear to have minor effects on life expectancy in industrialized countries, but that the effects of these changes are greater if they occur in conjunction with other major social and political changes.

## Abstract (de)

Um den Einfluss ökonomischer Bedingungen und Krisen auf die Sterblichkeit und ihre Vorhersagbarkeit in industrialisierten Ländern zu untersuchen, geben wir einerseits einen Einblick in bisherige Forschungsergebnisse und andererseits prognostizieren wir die Sterblichkeit in Spanien, Ungarn und Russland, drei Länder, die große Transformationsprozesse (inklusive ökonomischer und politischer Reformen) in den vergangenen Jahrzehnten erlebt haben. Die retrospektiven Sterblichkeitsprognosen von 1991 bis 2009 zeigen, dass unser Prognosemodell Veränderungen im Langzeittrend einfangen kann und dass die Abweichungen von der tatsächlich eingetroffenen Sterblichkeitsentwicklung häufig geringer sind als die anderer weit verbreiteter Modelle (wie z. B. das Modell von Lee und Carter). Zwei methodische Besonderheiten scheinen dafür (ganz besonders) verantwortlich zu sein: Erstens kann unser Modell die dynamische Verschiebung starker Sterblichkeitsverbesserungen von jungen zu hohen Altern über die Zeit einfangen und zweitens kann es wesentliche Veränderungen im Langzeittrend modellieren, indem es optional den extrapolierten Trend in einem Land um extrapolierte Trends ausgewählter Referenzländer ergänzt. Gleichwohl kann aber auch die Vorhersageleistung unseres Modells unter bestimmten Prognosebedingungen limitiert sein: Wenn sich z. B. der Sterblichkeitstrend sehr sprunghaft verändert (wie z. B. in Russland in der Zeit nach der Auflösung der Sowjetunion), dann scheint eine genaue Vorhersage eher von Zufall als von methodischer und demografischer Expertise abzuhängen. Die vorliegende Analyse deutet generell darauf hin, dass der alleinige Einfluss ökonomischer Veränderungen auf die Lebenserwartung bei Geburt in industrialisierten Ländern eher klein zu sein scheint, sich dieser aber erhöhen kann sobald er mit anderen sozialen und politischen Transformationsprozessen einhergeht.

## Introduction

**Fig. 1:**
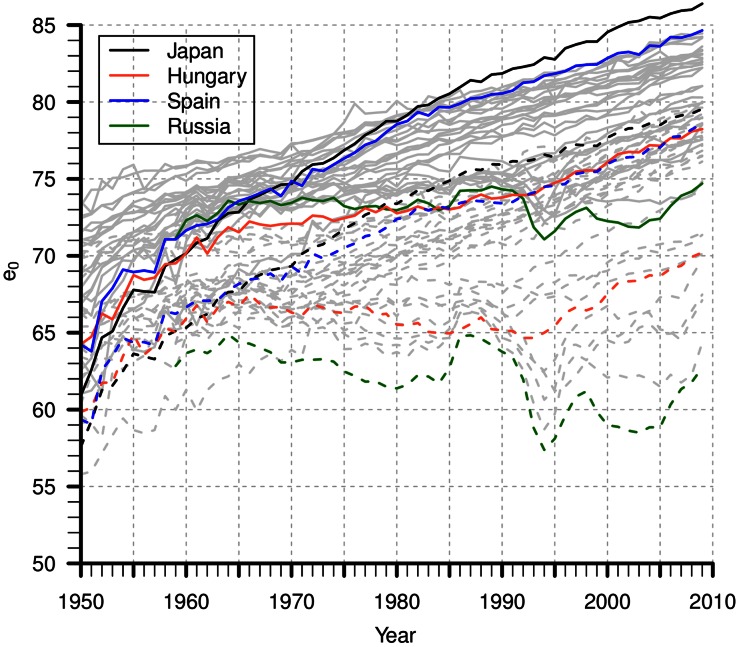
Observed life expectancy at birth (*e*_*0*_) for women *(solid lines)* and men *(dashed lines)* in 30 countries of the Human Mortality Database (2013) from 1950 to 2009. The trajectories for Japan, Hungary, Spain, and Russia are highlighted in *black, red, blue*, and *green*; respectively

Oeppen and Vaupel (2002) have shown that for well over a century female record life expectancy has been increasing by approximately 2.5 years per decade, or by about 6 h per day. However, as Fig. 1 illustrates, from 1950 to 2009 there have been substantial differences in the development of life expectancy for women (solid lines) and men (dashed lines) across the countries of Europe, as well as in Australia, Japan, and the United States. Moreover, while highly developed countries have generally seen steady increases in life expectancy in recent decades, the mortality patterns in many countries of central and Eastern Europe in the second half of the twentieth century have been irregular, as progress was interrupted by periods of stagnating and even decreasing life expectancy. For instance, in 2009 Japan (black) held the record for life expectancy, at 86.4 years for women and 79.6 years for men; while life expectancy in Russia (green) lagged far behind, at only 74.7 years for women and 62.73 years for men. Like Japan, Spain experienced a rapid increase in life expectancy between 1950 and 2009. Meanwhile, between 1965 and 1990, Hungary, like Russia, saw only small improvements in survival, and even registered increases in mortality. Yet unlike Russia, Hungary has been catching up to international trends since the early 1990s.

In light of these trends, the following question arises: Which factors affect mortality *per se*, and which factors had the greatest impact in the last 50 years? The scale of mortality fundamentally depends on both individual and structural factors. These individual factors include socioeconomic characteristics like age, sex, education, and occupation; and lifestyle choices such as diet, physical exercise, and smoking. Among the structural factors are conditions related to standard of living, access to education and health care, employment, and distribution of income and wealth. Although it is difficult to disentangle the influence of each of these factors on mortality, it is clear that their effects vary over time. Improvements in public health, such as water purification and hygiene, raised the standards of living in many developed countries up to the 1930s (Cutler et al. 2006). Socioeconomic conditions might play a larger role today.

In this article, we investigate the impact of economic changes on mortality and how the effects of these changes may become stronger in the presence of major political and social changes. We have chosen to focus on Spain, Russia, and Hungary; three developed countries which have recently undergone major political and economic transitions to, for example, democracy or capitalism. Such transitions typically differ in terms of their balance of political and economic reforms, their pace and severity, and their sustainability. While Spain moved directly from being governed by a military dictatorship to democratic rule in the 1970s (Mackenbach et al. 2013), the trajectories of political and economic reforms in countries affected by the dissolution of the Soviet Union—including Hungary and especially Russia—were less straightforward (Grigoriev et al. 2010). In central and eastern Europe, rising income inequality, a deteriorating social safety net, and increasing material hardship (Bobak et al. 1998 2000) might have led to elevated levels of psychosocial stress. Mortality in these countries, and especially in Russia, also increased due to excessive alcohol consumption, accidents, and cardiovascular diseases (Leon 2007; Shkolnikov and Cornia 2000; Shkolnikov et al. 2004).

Forecasting mortality is difficult *per se* since we cannot know with any certainty what will happen in the future. But generating such forecasts becomes particularly challenging if mortality develops irregularly, as it typically does in countries after major transformation processes. In this article, we employ our mortality forecasting model (Bohk and Rau 2014a), which is a flexible framework that can be applied to forecast regular as well as irregular mortality developments. For instance, our model can capture substantial changes in long-term trends by optionally complementing extrapolated mortality trends in a country of interest with those of selected reference countries. To test the forecasting performance of our model under challenging conditions, we have generated (retrospective and prospective) mortality forecasts for Spain, Hungary, and Russia. We then assessed the forecasts of our model by comparing the results with (1) the observed mortality development (at least in the retrospective setting) as well as with (2) the results of other well-accepted mortality forecasting models; in this case, with the model of Lee and Carter (1992) and its coherent variant of Li and Lee (2005).

## Economic impact on mortality

**Fig. 2:**
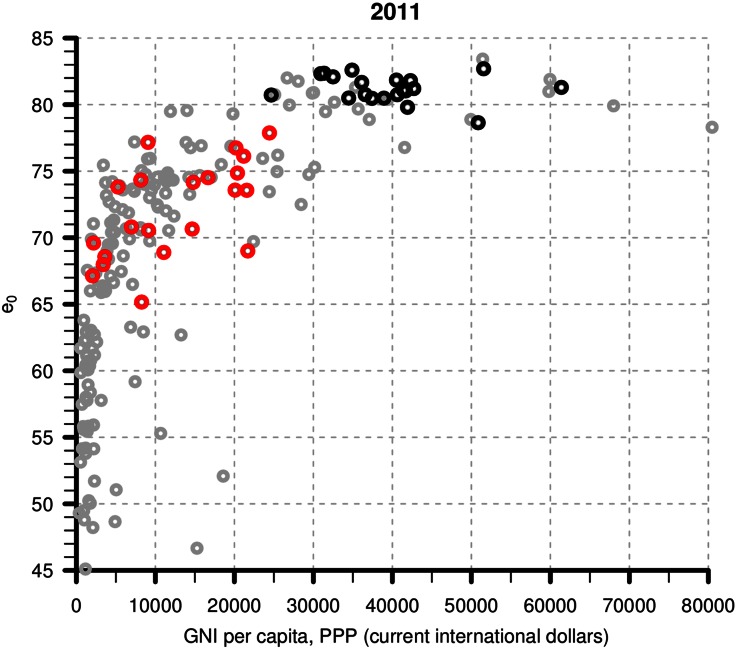
Gross National Income (*GNI*) per capita, PPP (*in current international dollars*) and life expectancy at birth (*e*_*0*_) for 236 countries in 2011. We depict highly developed countries from the Human Mortality Database (2013) in *black* and central and eastern European countries (that formerly belong to the Warsaw Pact) in *red*. Data are from the International Comparison Program database (World Bank 2013)

Preston (1975, 2007) analyzed the impact of economic conditions on the level of mortality, assuming that higher income per capita allows people to spend more money on health-related goods and services like food, housing, leisure, and education. He applied a logistic model to describe the relationship between income per capita and life expectancy at birth for many countries all over the world in three periods: i.e., 1900, 1930, and 1960. He found that countries with a higher income per capita also have a higher life expectancy. Although Preston’s main focus was on economic conditions, he also observed that progress in life expectancy was influenced by factors like living standards, vaccines, and antibiotics, at least in the first half of the twentieth century. Figure 2 depicts this global relationship between higher gross national incomes and higher life expectancy for 236 countries in 2011. Comparing economic growth across countries and linking it to mortality is, however, often fraught with difficulties. For instance, Nesporova (2000) and Fleming et al. (2000) emphasized the role of the shadow (or informal) economy, which may have accounted for around 25 % of all economic activity in transition countries like Hungary and Russia in the early 1990s; whereas Sen (1998) has observed that increases in life expectancy may be attributable not only to GNP per capita, but also to the availability of public health services and to poverty reduction. To compare levels of income across countries, we have nonetheless chosen to apply the gross national income converted to international dollars using purchasing power parity rates (GNI per capita, PPP). For instance, in 2011 the highly developed country of Norway had a relatively high GNI per capita and a PPP rate of approximately 61,390 international dollars. Norway also had, at 81 years, a life expectancy that was 12 years higher than that of Russia, a country which had a relatively low GNI per capita and a PPP rate of approximately 21,700 international dollars. Indeed, Fig. 2 shows that countries with a higher GNI have a higher life expectancy than countries with a lower GNI. But Fig. 2 also shows that this global relationship is curvilinear, which implies that the economic impact weakens once a certain level of life expectancy has been reached (“inelasticity”).

Since our focus is on industrialized countries, we review studies that investigate the (weakening) influence of economic conditions on further gains in life expectancy from different perspectives. For instance, Wilkinson (1990, 1992) showed that socioeconomic inequalities—as indicated by, for example, relative poverty—influence public health more strongly than economic growth (via increasing income per capita). These findings are in line with those of previous research by Rodgers (1979), who stressed the positive association between high levels of socioeconomic inequality and elevated mortality. Christensen et al. (2009) and Lee and Mason (2011) gave demographic explanations for the weakening influence of economic conditions on the progress of life expectancy in developed countries over time: while past mortality reductions resulted in a larger share of people of working age, and therefore in economic growth and increasing GDP per capita; recent mortality reductions have resulted in a higher share of people of retirement age, and therefore in less economic growth and smaller increases in GDP per capita. Prolonged lives, coupled with a larger share of older people in the population, tend (1) to reverse the direction of intergenerational transfers (from elderly to young), and (2) to increase the pressure on the social welfare system, since fewer people of working age have to support more and more retired elderly people. The redistribution of work (including a rise in the retirement age), as well as increased productivity (including investments in health-related education, medical technology, and innovations) might be two strategies for overcoming the socioeconomic consequences of this demographic transition. Whereas these studies sought to attribute the weakening economic impact on mortality to socioeconomic inequality and demographic aging from a period perspective, other scholars have examined the extent to which mortality in later life can be affected by economic conditions early in life from a cohort perspective. For example, van den Berg et al. (2006, 2009) used data for Dutch and Danish birth cohorts in the nineteenth century to show that individuals born in a recession have fewer years of remaining life expectancy at advanced ages than individuals born in a boom. More recent studies have also presented evidence that economic conditions in early life affect particular kinds of health conditions at advanced ages, especially those related to cardiovascular health and cognitive abilities (Doblhammer et al. 2013; van den Berg et al. 2011). According to these studies, individuals born in a boom have significantly better cognitive abilities and are less likely to suffer from cardiovascular diseases than individuals born during a recession. Barker (1990, 2007) also pointed out that not only neonatal and postnatal, but also prenatal (or fetal) conditions (such as undernutrition *in utero*) might contribute substantially to the development of coronary heart diseases in later life.

Since some countries experienced relatively large gains in life expectancy even with little or no economic growth, Cutler et al. (2006) argued that economic conditions play only a marginal role in mortality improvements. They instead attributed these improvements primarily to advances in health-related technologies, which may influence both health and income. For instance, Chinese life expectancy increased rapidly between 1950 and 1970, despite the low levels of growth in income per capita in China during that period (Wang 2011). These substantial survival improvements have mainly been attributed to factors like increased economic equality (due to a socialist welfare system), improved public health care, and the spread of modern health and medical knowledge (Wang 2011, p. 177). However, other scholars have questioned the association between income inequality and life expectancy, pointing out that the results strongly depend on the selected countries, time periods, and sources of income data (Lynch and Smith 2002; Mackenbach 2002). For instance, Lynch et al. (2001), using data of better quality, found little (or no) evidence for the association between income inequality and mortality differences in 16 wealthy countries.

Crises are processes that abruptly transform the social, economic, and/or political structure of a population. Because these transitions are often unexpected and allow people little time to prepare, they tend to induce a sense of insecurity. Such crises occurred, for example, in Spain, Portugal, and Greece in the 1970s; and in the Warsaw Pact states in the 1990s. Moreover, in the wake of the financial crisis of the late 2000s, similar crises are currently unfolding in countries like Greece and Spain. Mackenbach et al. (2013) analyzed the impact of democratization on life expectancy: in Spain, Portugal, and Greece, survival improvements were immediately recognizable after the transformation from military dictatorship to full democracy in the 1970s; whereas in the 1990s in the countries of the former Soviet Union and its satellite states, it took longer for survival improvements to emerge because the transformation affected not only political, but also economic and social dimensions. While the Baltic countries successively democratized and joined the European Union, other countries like the Russian Federation experienced a kind of reversion to the previous (political and economic) conditions (Mackenbach et al. 2013). The diverse facets of the mortality crisis in these transitional countries have been described in detail in Cornia and Panacciá (2000a, b), who have provided relevant background information on the region as a whole, as well as on individual countries. In addition, they have explained and discussed the causative conditions that most affected vulnerable people, as well as the policy approaches used to tackle such transformation processes. Meslé (2004) and Grigoriev et al. (2010) have also pointed out that the effects of the dissolution of the former Soviet Union varied across central and eastern European countries depending on their initial conditions, including their industrial structure and their levels of income and human capital (Popov 2007, 2009). In the early 1990s, radical economic and political reforms led to substantial increases in mortality in Russia and Lithuania, whereas the more modest economic and political changes in Belarus during this period resulted in smaller increases in mortality (Grigoriev et al. 2010). It is also striking that the mortality increases in Russia mainly occurred among men of working age, many of whom were unemployed, poorly educated, unmarried, and living in urban areas. These deaths were largely due to excessive alcohol consumption, accidents, and cardiovascular diseases; and were less likely to occur among the very young and the very old, the groups who are usually most affected by such crises (Leon 2007; Shkolnikov and Cornia 2000; Shkolnikov et al. 2004). The well-being of a population in terms of their economic prospects and health status depends not only on the speed of economic liberalization and democratization, direct foreign investment (including mass privatization (Stuckler et al. 2009)), and free trade, but also on the willingness of policy-makers and the polity to pursue strategies that meet the country’s specific requirements (Popov 2007, 2009).

Even today, health and mortality conditions might be influenced by financial (and economic) crises. For instance, Bernal et al. (2013) and Roca et al. (2013) have provided evidence that suicides and mental disorders have increased in Spain since the beginning of the financial crisis of the late 2000s, and that people of working ages have been hit particularly hard by very large increases in unemployment. According to data from the UN (The United Nations Statistics Division (UNSD) of the Department of Economic and Social Affairs (DESA) 2014), the unemployment rate in Spain increased for both men and women from 9.63 % in the first quarter in 2008 to 26.03 % in the fourth quarter in 2013. Kentikelenis et al. (2011, 2012) showed similar effects for Greece, reporting not only an increase in suicide, but also in psychiatric morbidity. The cross-sectional studies of Bobak et al. (1998, 2000) have provided empirical evidence for Russia that following the abrupt transition to democracy and capitalism in the 1990s, rising income inequality, a deteriorating social safety net, and increasing levels of material hardship have resulted in greater psychosocial stress among the population; and that this stress in turn led to higher levels of morbidity and premature deaths. Thus, it appears that a perceived loss of control over one’s life, especially when coupled with material deprivation, has strong effects on self-reported health.

Although the financial crisis of the late 2000s resulted in increased levels of economic insecurity in many countries, not all countries and not all individuals were equally affected. Since young adults appear to be particularly vulnerable to rising unemployment rates, healthy and mobile young people may be motivated to emigrate to countries which offer them better job opportunities. Receiving countries such as Germany might benefit from an influx of young, healthy, and highly educated immigrants; while sending countries such as Spain could suffer from this brain drain. These migration flows could also have effects on life expectancy: while receiving countries might experience gains in life expectancy due to the healthy migrant effect, the sending countries might experience decreases in life expectancy.

## Methodological considerations on mortality forecasting

**Fig. 3:**
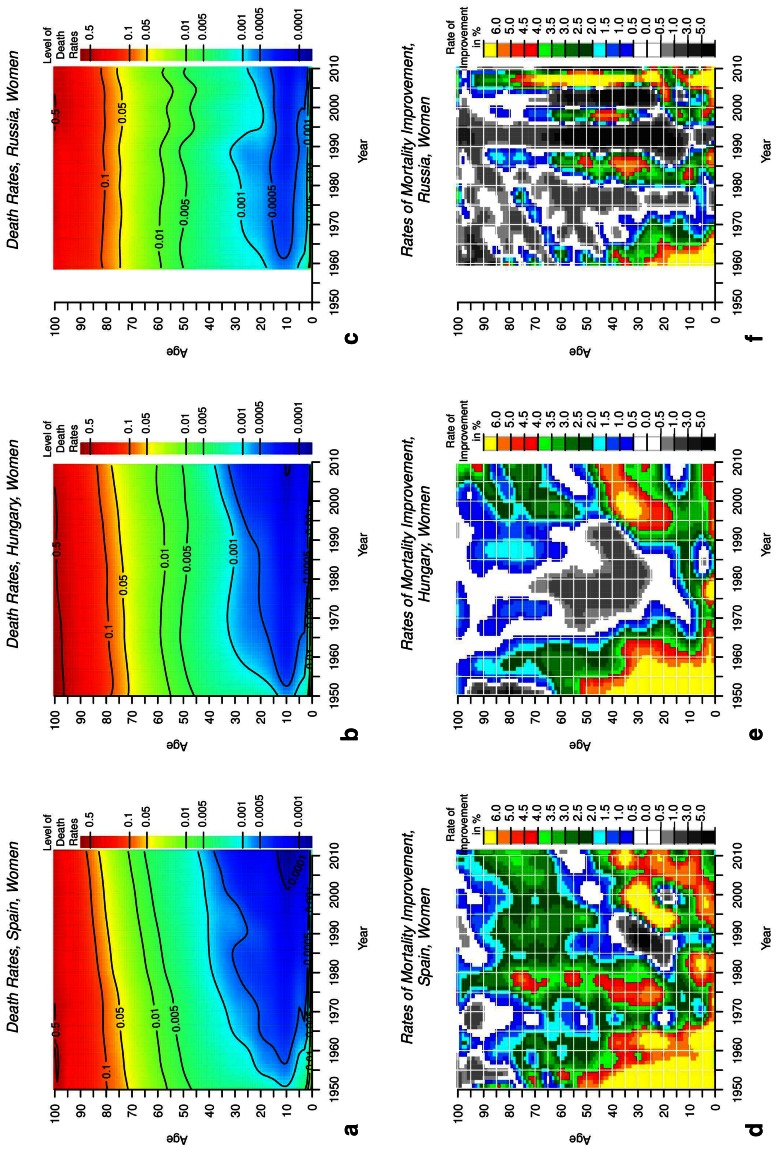
(*Smoothed*) Death rates (*upper panel*) and their corresponding rates of mortality improvement in % *(lower panel)* for women in Spain (*left*; **a, d**), Hungary (*center*; **b, e**), and Russia (*right*; **c, f**) from 1950 to 2009

**Fig. 4:**
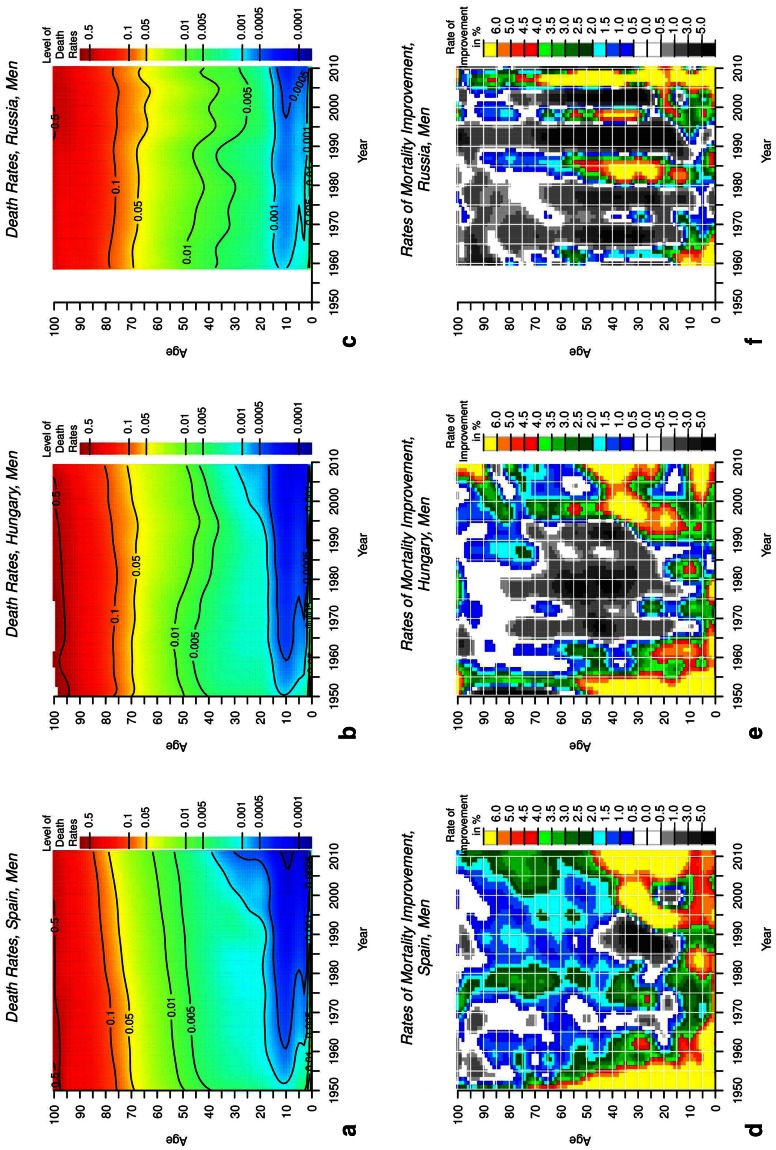
(*Smoothed*) Death rates (*upper panel*) and their corresponding rates of mortality improvement in % (*lower panel*) for men in Spain (*left*; **a, d**), Hungary (*center*; **b, e**), and Russia (*right*; **c, f**) from 1950 to 2009

The fact that (record) human lifespans are continuously expanding suggests that mortality tends to evolve according to a fairly regular pattern, even though the substantial differences that emerge both between countries and within countries over time seem to imply that mortality rates are volatile (see Fig. 1). Analyzing mortality disaggregated by age and sex reinforces this latter impression, since it reveals changes in age-specific mortality that differ in their direction as well as in their intensity over time. Figures 3 and 4 illustrate the death rates (top row), as well as their rates of improvement (bottom row); i.e., the time derivative of the age-specific death rates, in percent for women and men in Spain (left), Hungary (center), and Russia (right) from 1950 to 2009 on so-called Lexis surfaces by calendar time and age. Since a 1 year increase in age is equivalent to a 1year increase in time, each birth cohort is represented by a 45-degree line. Gradually declining death rates are indicated by successively increasing contour lines, which represent how a certain mortality level (of, for instance, 0.01) dynamically shifts to older ages with time. Such mortality declines are caused by survival improvements, which we depict as the rates of mortality improvement. Strong survival improvements (of more than 4 %) are shown in red and yellow; while minor and moderate survival improvements (of more than 0.5 % and less than 4 %) are shown in blue and green. Stagnating mortality (between − 0.5 and + 0.5 %) is depicted in white, and increasing mortality (up to − 5 %) is illustrated in gray and black. The age-specific death rates—and, to an even greater degree, their corresponding rates of improvement—in Spain, Hungary, and Russia clearly show that the developments in mortality in these countries have been volatile, often as a result of the period effects of political, social and/or economic crises:

### Spain

Relative to other countries, Spain experienced some of the largest gains in life expectancy between 1950 and 2009 (see Fig. 1). These gains occurred because of relatively large reductions in mortality at different ages over time. Particularly remarkable are the relatively moderate and strong survival improvements (green and red rates of mortality improvement in Figs. 3 and 4) at almost all ages in the 1960s, a period in which Spain experienced an unprecedented level of economic growth, also known as the Spanish miracle; as well as in the 1970s, a period in which Spain transitioned from Franco’s dictatorship to democracy.

### Hungary

Hungary gained far fewer additional years of life than Spain between 1950 and 2009, and experienced periods of stagnating and slightly decreasing life expectancy (at least for men) between 1960 and 1995; this pattern is similar to those observed in many central and eastern European countries, and is often attributed to an ineffective health care system and unhealthy lifestyles (regarding alcohol consumption and tobacco use). This trend of increasing mortality reversed about 5 years after the dissolution of the Soviet Union, and Hungarian life expectancy has since caught up to international trends. Increased investments in the health care system, including improved medical treatments and prevention efforts, along with declines in cardiovascular mortality appear to be the driving forces behind these recent gains (Balogh et al. 2010; Meslé 2004), and differentiate Hungary from Russia (Shkolnikov et al. 2004).

### Russia

The development of Russian life expectancy between 1950 and 2009 is characterized by considerable volatility, including periods of decreasing, stagnating, and increasing mortality. In contrast to Hungary, Russia experienced not just a slight, but a sharp increase in mortality in the years following the dissolution of the Soviet Union among people of all ages (gray and black rates of mortality improvement). Russian life expectancy did not start to increase again until 2005, almost 10 years after Hungary began to register improvements. Currently, life expectancy levels in Russia continue to lag far behind those of other European countries. According to Shkolnikov et al. (2004), the persistence of this large gap in life expectancy until the mid-2000s is attributable to many decades of relatively low levels of investment and a lack of technological innovation in the health care system; along with excessive alcohol consumption, tobacco use, and relatively high levels of psychosocial stress, which are linked to a high rate of premature death. Although improvements in the health care system have since been made and the numbers of premature (cardiovascular and external) deaths have declined since 2005, Shkolnikov et al. (2013) pointed out that it is still unclear whether the recent progress in Russian life expectancy can be sustained in the future, and whether these positive developments mean that Russia is finally catching up to international trends.

In spite of these ups and downs, a regular pattern of mortality decline is clearly visible in all three countries. This pattern is most evident in the dynamic shift in mortality from younger to older ages, the trend which has contributed the most to improvements in life expectancy. While political, social, and/or economic crises have slowed this development for certain periods of time, they have not changed the general direction. Since these relatively large survival improvements have not yet affected the oldest-old in any of these three countries, we expect this dynamic age shift will continue in the future, leading to further gains in life expectancy.

Given these diverse mortality developments, a model used to generate precise and plausible mortality forecasts must meet at least three criteria. First, a mortality forecasting approach should be able to model dynamic age shifts (in survival improvements from younger to older ages). Second, it should be able to model fundamental changes in long-term trends (due to substantial transformation processes). Third, it should be able to incorporate how probable each potential mortality development actually is. If a model meets all of these criteria, it is technically/methodologically equipped to forecast regular as well as irregular mortality developments. This is, however, no guarantee of accurate mortality forecasts, since some events are (and will be) unexpected.

Today, providing probability statements for mortality forecasts is considered the state-of-the-art. Modeling dynamic age shifts in mortality reductions and fundamental changes in long-term trends remains challenging. Many mortality forecasting approaches simply extrapolate past trends, and are therefore severely limited when it comes to including dynamic age shifts or turning points in long-term trends. For instance, the widely applied Lee-Carter model (Lee and Carter 1992) extrapolates death rates by age on the logarithmic scale. Although this approach can model improving survival, it assumes that relative mortality changes among ages and among adjacent years remain constant over time. As these strong constraints are implausible, large errors in the forecasts generated are likely, especially in the long run. Mitchell (2013) and Haberman and Renshaw (2012) tried to overcome this inflexibility by taking (and extending) the predictor structure of the original Lee-Carter model in order to forecast the rates of mortality improvement instead of the death rates. This procedure allowed them to capture relative mortality changes more flexibly and to model dynamic age shifts, which is one reason why we also have chosen to use the rates of mortality improvement in our mortality forecasting model.

Countries like Spain, Hungary, and Russia experienced mortality developments that were interrupted by periods of sharp mortality declines or survival improvements due to major transformation processes. Forecasting such unsteady mortality developments is particularly challenging, because turning points in long-term trends are often unexpected and do not naturally emerge from the past; instead, these turning points tend to occur abruptly due to radical changes in essential areas of a society. So-called *coherent* approaches might allow us to capture such long-term trends. These approaches jointly forecast mortality trends among multiple (sub)populations. For instance, Li and Lee (2005) and Cairns et al. (2011) have suggested the sharing of mortality trends among a group of countries that are connected through geographical, social, economic, political, or cultural proximity. If a country experiences a smaller increase in life expectancy than the group of countries to which it is similar, a coherent model might forecast a larger increase in life expectancy for this country than would otherwise have been generated, based on the assumption that the country will likely catch up to the broader trend. Hence, a coherent forecast can alter long-term trends due to the inclusion of information from other related countries. This is a reason why our model also allows us to use the mortality trends of selected reference countries as additional information.

The above-mentioned requirements served as a basis for the development of our model (Bohk and Rau 2014a), which can be used to generate probabilistic forecasts for populations experiencing regular as well as irregular mortality trajectories. Instead of using a simple regression model with dummies accounting for crises, we combined recently developed concepts to forecast mortality: our model can capture dynamic age shifts in mortality reductions via rates of mortality improvement, and it can capture major changes in long-term trends by optionally complementing mortality trends in a country of interest with those of selected reference countries. We use Bayesian inference and simulation-based Markov chain Monte Carlo methods to process our hierarchical models, which provide a predictive outcome distribution with probability statements for all possible mortality developments in order to capture and quantify forecasting uncertainty.

## Mortality forecasting in practice

We have already applied our model successfully in retrospective and prospective mortality forecast designs for Great Britain and Denmark (Bohk and Rau 2014a) as well as for Italy, Spain, Sweden, and West Germany (Bohk and Rau 2014b). However, since the purpose of this paper is to show how our model can be used to forecast irregular mortality developments, here we have generated mortality forecasts for Spain, Hungary, and Russia.

### Retrospective mortality forecasts

In the retrospective setting, we forecast mortality by single age and sex from 1991 to 2009, given data from 1965 to 1990. We determine that the base period (1965–1990) ends at the point in time of the dissolution of the Soviet Union, which also marked the start of political, social, and economic crises in Hungary and Russia. Hence, these retrospective forecasts can show how forecasting models are able to capture major changes in long-term mortality trends: in the case of Hungary, an upward trend in life expectancy; and in the case of Russia, a downward trend in life expectancy (at least until 2005). To enable comparability, we use the same base period (1965–1990) for Spain.

To assess the forecasting performance of our model, we use forecast errors that quantify the difference between forecasted and observed mortality data; we then use these forecast errors to compare the forecasting performance of our model with the performances of other renowned models, in particular with the original Lee-Carter model (1992) and its coherent variant (Li and Lee 2005). To generate the retrospective mortality forecasts with the Lee-Carter model (1992), we use its freely available implementation, written by Timothy Miller. For the forecasts with the coherent Lee-Carter variant of Li and Lee (2005), we use the web-based platform *LCFIT*.

Since both our model and the coherent Lee-Carter model can jointly forecast the mortality trends of multiple populations, we use Portugal as a reference country for Spain, eastern and western Germany as reference countries for Hungary, and Belarus as a reference country for Russia.

**Fig. 5:**
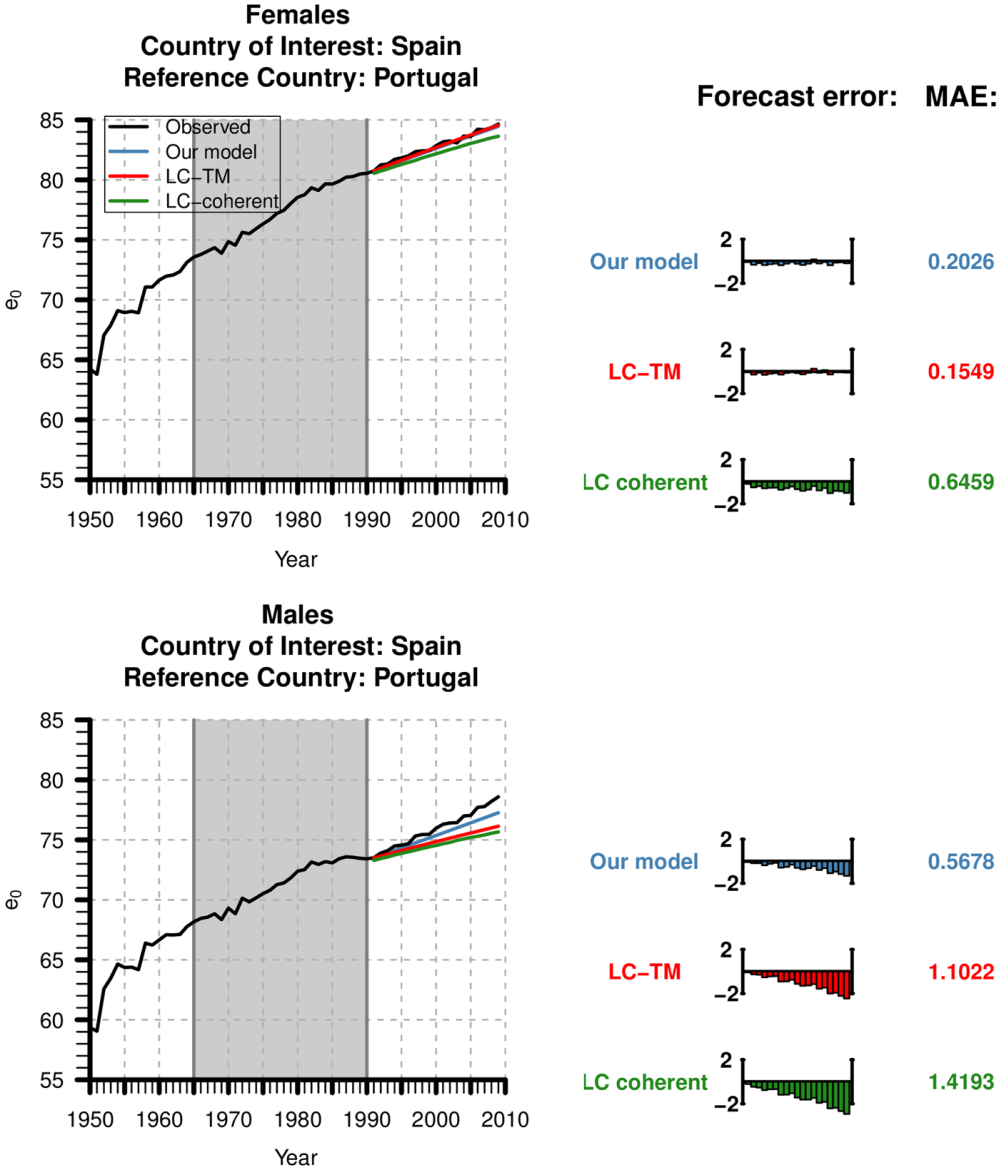
*Left*: Observed life expectancy at birth *(black line)* as well as its retrospective forecasts of our model *(blue line)*, of the original Lee-Carter model *(red line)* and of its coherent variant *(green line)* for women *(top)* and men *(bottom)* in Spain. We take data from 1965 to 1990 *(gray square)* to forecast mortality from 1991 to 2009. *Center*: Annual forecast errors for each model. *Error* indicates that forecasting models were run, but generated an error message. *Right*: Mean of the absolute forecast errors (MAE) over all forecast years for each model

**Fig. 6:**
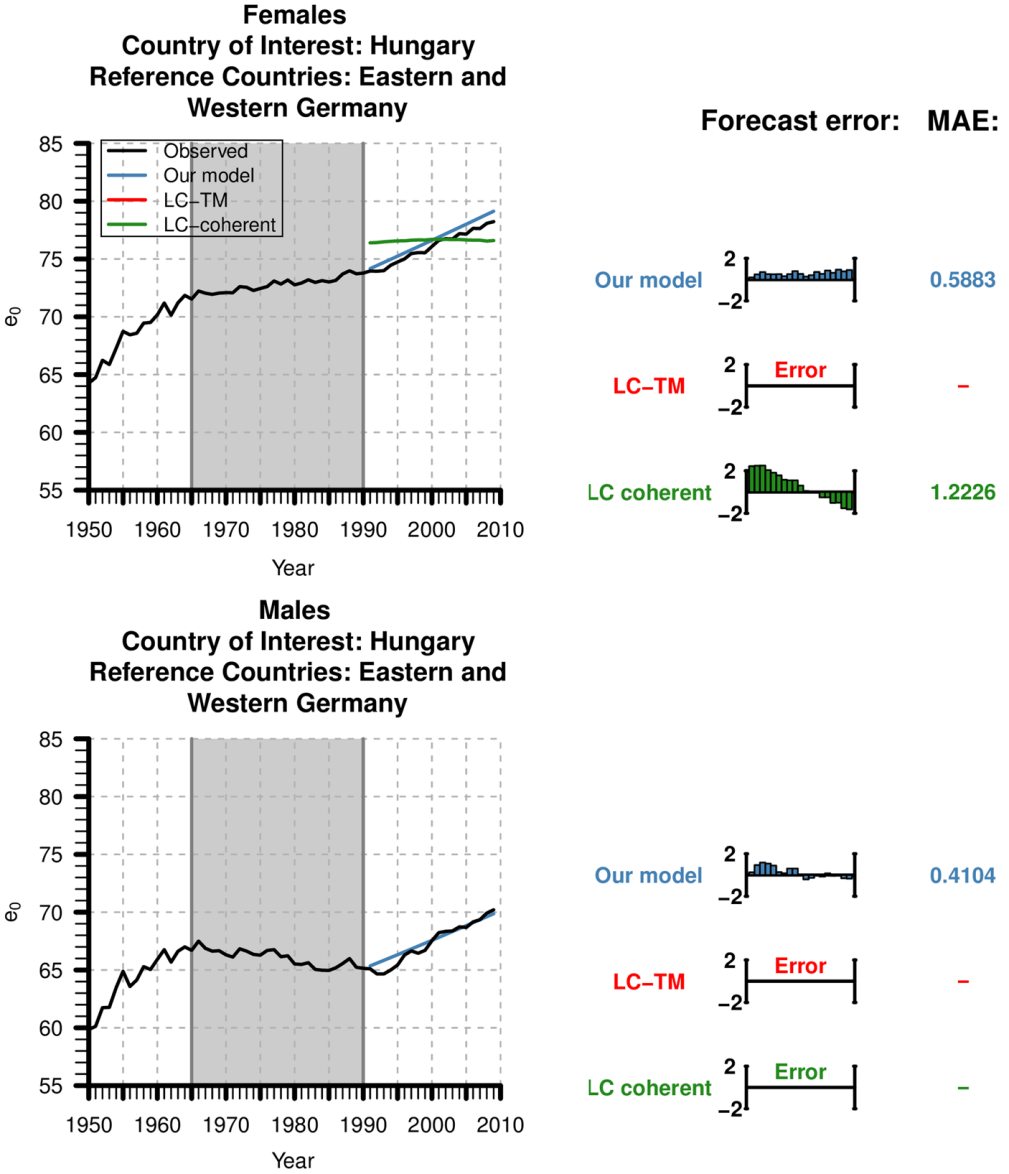
*Left*: Observed life expectancy at birth *(black line)* as well as its retrospective forecasts of our model *(blue line)*, of the original Lee-Carter model *(red line)* and of its coherent variant *(green line)* for women *(top)* and men *(bottom)* in Hungary. We take data from 1965 to 1990 *(gray square)* to forecast mortality from 1991 to 2009. *Center*: Annual forecast errors for each model. *Error* indicates that forecasting models were run, but generated an error message. *Right*: Mean of the absolute forecast errors (MAE) over all forecast years for each model

**Fig. 7:**
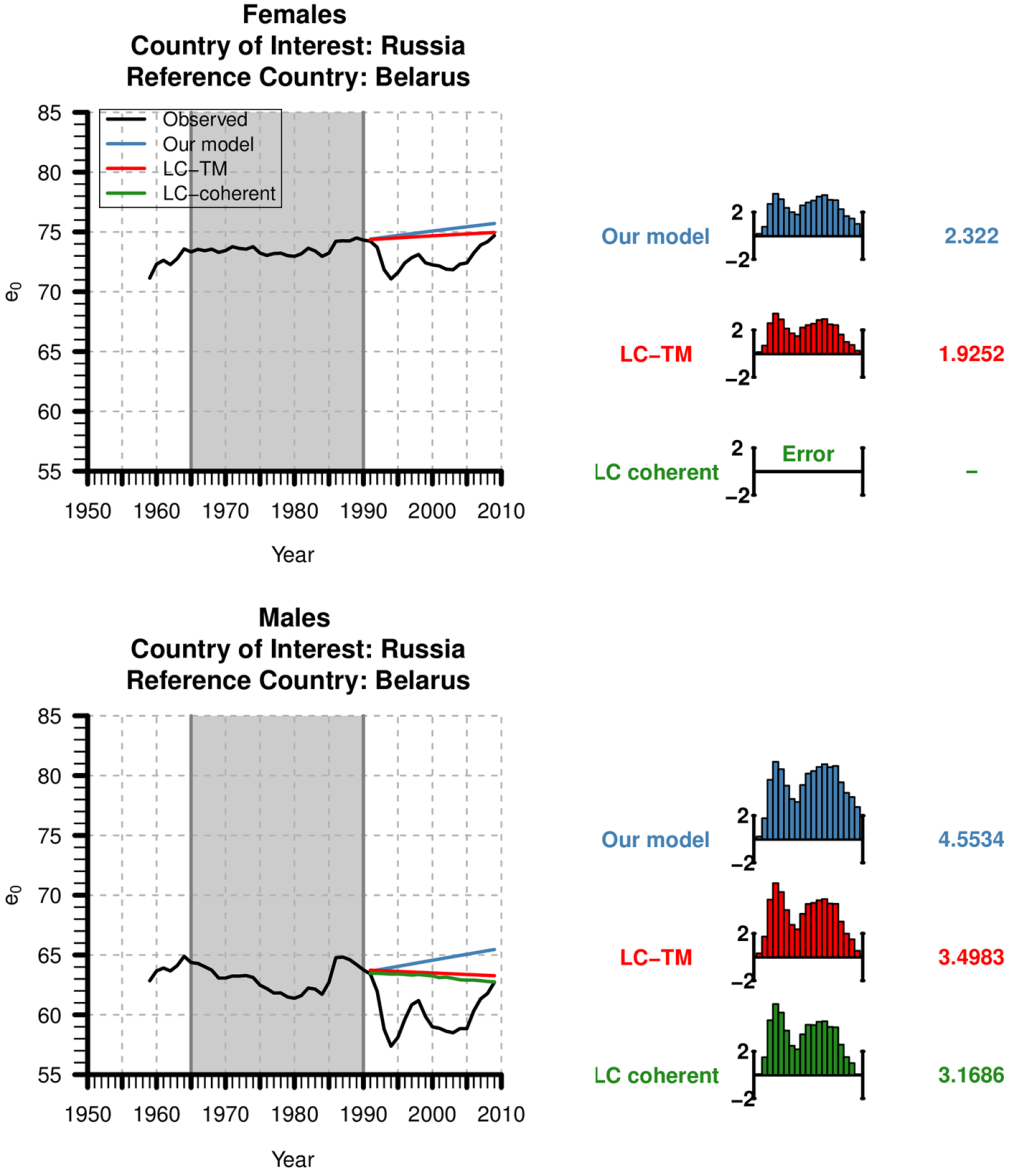
*Left*: Observed life expectancy at birth *(black line)* as well as its retrospective forecasts of our model *(blue line)*, of the original Lee-Carter model *(red line)* and of its coherent variant *(green line)* for women *(top)* and men *(bottom)* in Russia. We take data from 1965 to 1990 *(gray square)* to forecast mortality from 1991 to 2009. *Center*: Annual forecast errors for each model. *Error* indicates that forecasting models were run, but generated an error message. *Right*: Mean of the absolute forecast errors (MAE) over all forecast years for each model

Figures 5, 6 and 7 depict the observed (black line) and forecasted life expectancy of our model (blue line), of the original Lee-Carter model (red line), and of its coherent variant (green line) for both sexes in Spain, Hungary, and Russia. In addition, we assess the retrospective mortality forecasts with forecast errors and mean absolute errors (MAEs), which are also depicted in those figures. Forecast errors quantify the difference between forecasted and observed life expectancy in every year; they measure bias, since they can be greater or less than zero, indicating whether a forecast systematically over- or underestimates observed life expectancy. In contrast, the MAEs measure accuracy, since they are the mean of the absolute forecast errors over all years, only taking the number of deviations into account, irrespective of whether they are positive or negative. While the models perform almost equally well in the case of Spain, their performance differs considerably in the cases of Hungary and Russia.

#### Spain

The female mortality forecasts of our model and of the original Lee-Carter model fluctuate narrowly around the observed life expectancy in Spain, resulting in small MAEs that range from 0.15 to 0.2. Only the coherent Lee-Carter model slightly underestimates female life expectancy, a fact that is indicated by negative forecast errors and a slightly larger MAE of 0.64. For men, all of the models underestimate the sharp increase in observed life expectancy from 1990 onward, but our model generates a smaller MAE, of 0.56, than the Lee-Carter model and its coherent variant, which produce MAEs of 1.10 and 1.41, respectively. Since the long-term mortality trend did not change substantially among women in the forecast years, all of the models can capture future developments for women quite well. Small difficulties appear only among men, for whom the upward trend in life expectancy intensifies/accelerates in the forecast years.

#### Hungary

While the forecasts of our model reflect the actual development of Hungarian life expectancy for women and men with only small deviations, the other models (1) generate larger forecast errors or (2) have problems forecasting Hungarian mortality at all. For instance, the female forecast errors of the coherent Lee-Carter model gradually decline from + 2.4 in 1991 to − 1.6 in 2009, resulting in a MAE of 1.22. The fact that there is already a relatively large deviation in the jump-off year indicates that the coherent model has problems pooling countries like Hungary with countries like eastern and western Germany, which have quite different mortality levels. This also demonstrates that our model uses mortality information from other countries in a similar, but still different way than the coherent Lee-Carter model: while our model uses mortality *trends* of other countries to model (successively evolving) major changes in long-term trends for a country of interest, the coherent Lee-Carter model jointly forecasts the mortality of populations with similar mortality *levels* (due to, for example, cultural proximity). Forecasting Hungarian mortality is especially challenging among men, since they experienced a radical change around the jump-off year (due to the dissolution of the Soviet Union): after declining for many years (in the base period), male life expectancy starts to increase sharply in the forecast years. This abrupt shift caused severe estimation problems for some models, including the original Lee-Carter model.

#### Russia

**Fig. 8:**
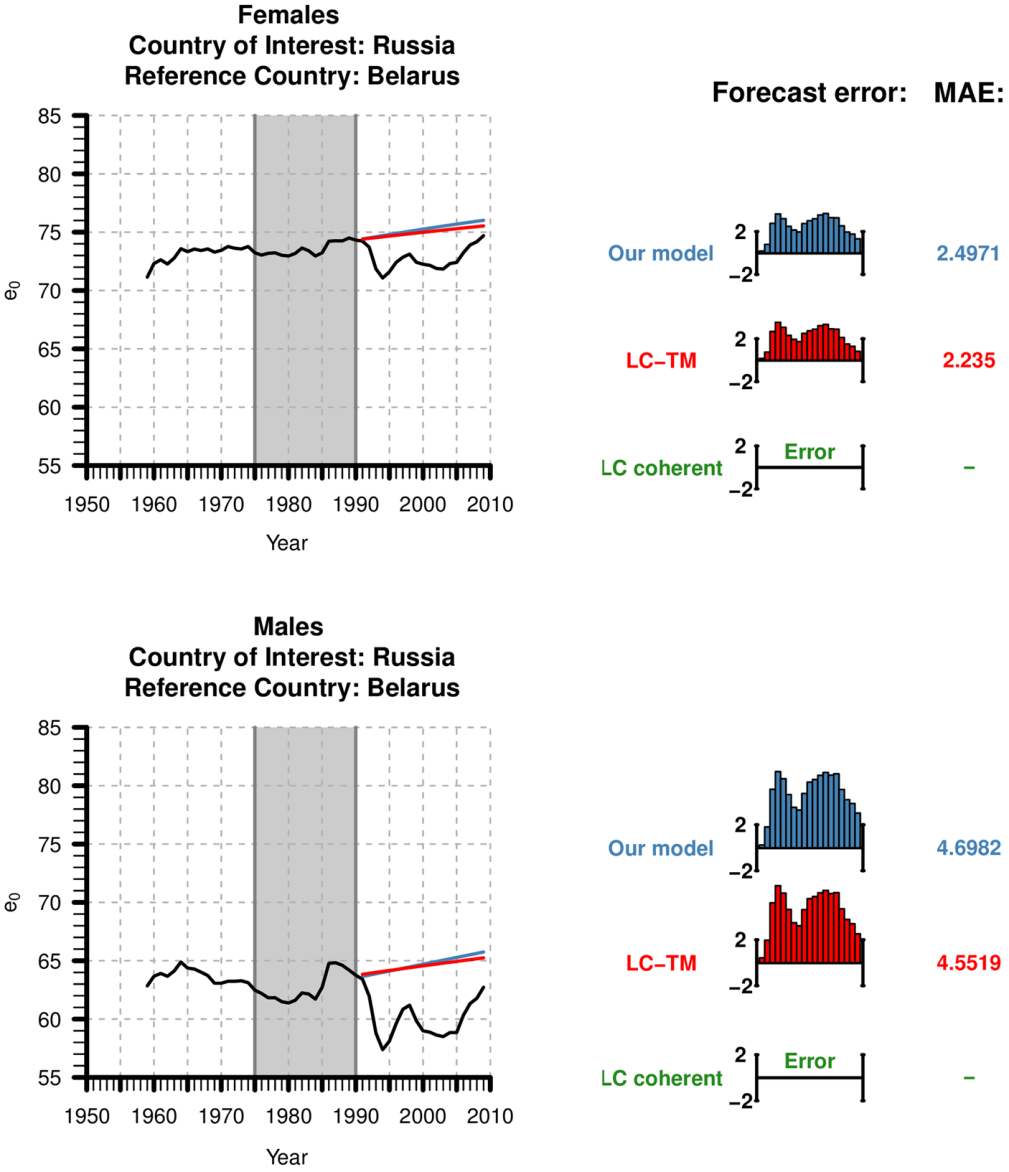
*Left*: Observed life expectancy at birth *(black line)* as well as its retrospective forecasts of our model *(blue line)*, of the original Lee-Carter model *(red line)* and of its coherent variant *(green line)* for women *(top)* and men *(bottom)* in Russia. We take data from 1975 to 1990 *(gray square)* in order to forecast mortality from 1991 to 2009. *Center*: Forecast errors; i.e., differences between forecasted and observed life expectancy, for each forecast year and model. *Error* indicates that forecasting models have been run, but have generated an error message. *Right*: Mean of the absolute forecast errors (*MAE*) over all of the forecast years for each forecast model

Since the development of Russian life expectancy was extremely volatile after the dissolution of the Soviet Union, including periods of sharp declines and smaller increases, all of the models generated relatively large forecast errors, overestimating the real development and resulting in MAEs that ranged from 1.9 to 2.3 for women and from 3.1 to 4.5 for men. Although our model overestimates Russian life expectancy to a greater extent than the other models, it is important to note that the selection of the base period has a large impact in this forecast setting. To show how sensitive the results are to the chosen base period, Fig. 8 depicts retrospective mortality forecasts, based on data from 1975 to 1990 (rather than 1965 to 1990). It is clear that both our model and the Lee-Carter model overestimate Russian life expectancy, but now they do so to a similar extent, since the MAEs of both models range from 2.2 to 2.4 for women and from 4.5 to 4.6 for men. Hence, extremely volatile mortality developments—like those observed among Russian males after the dissolution of the Soviet Union—are difficult to predict at all, and it seems that generating an accurate forecast depends more on luck than on methodology and expertise.

### Prospective mortality forecasts

**Fig. 9:**
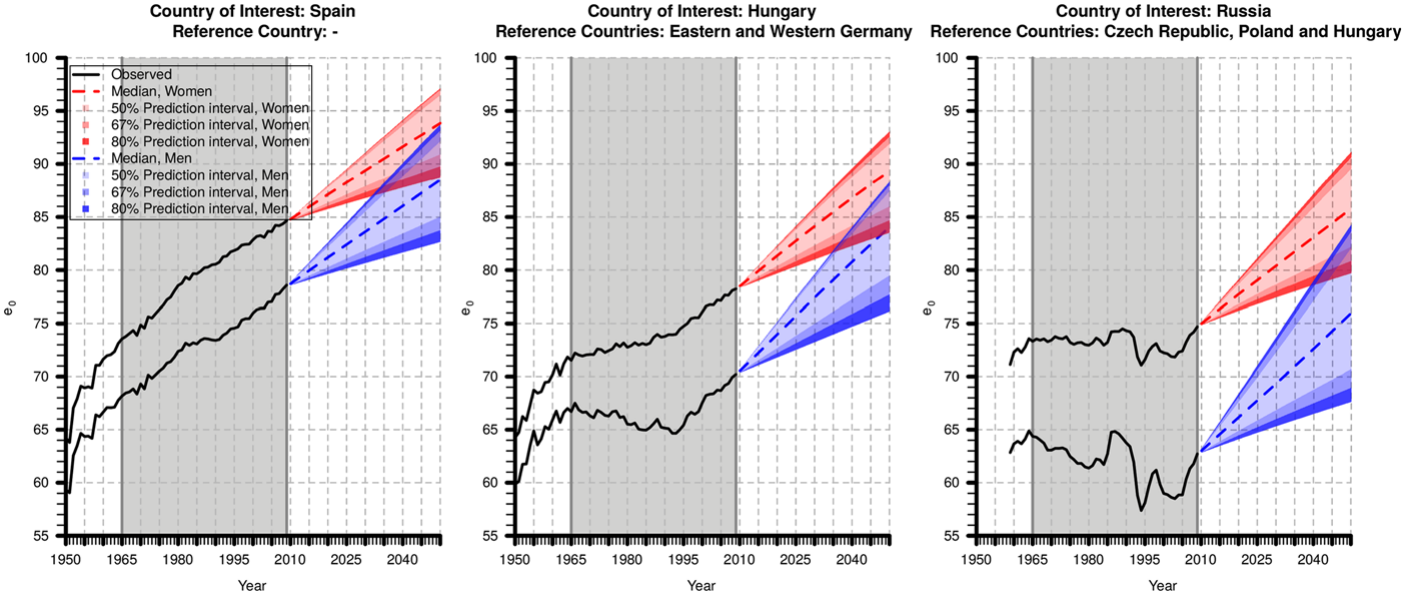
Observed *(black)* and forecasted life expectancy at birth of our model for women *(red)* and men *(blue)* in Spain *(left)*, Hungary *(center)*, and Russia *(right)*. We take data from 1965 to 2009 *(gray square)* in order to forecast mortality from 2010 to 2050. The width of the 80, 67, and 50 % prediction intervals—which gradually lighten as they move toward the median point estimates *(dashed lines)*—gives an impression of how uncertain the future development of mortality is

In the prospective setting, we employ our model to forecast mortality from 2010 to 2050, based on data from 1965 to 2009. We use (1) no reference country for Spain; (2) eastern and western Germany as reference countries for Hungary; and (3) the Czech Republic, Poland, and Hungary as reference countries for Russia, based on the assumption that Hungary and Russia are likely to catch up to international trends. We also expect that Hungary is likely to progress faster than Russia due to its greater political and economic proximity to highly developed countries in the European Union. Figure 9 and Table 1 depict the median point estimates, as well as the 50, 67, 80, and 95 % prediction intervals for female and male life expectancy in Spain, Hungary, and Russia from 2010 to 2050.

#### Median point estimates.

Our model clearly forecasts increasing life expectancy for both sexes in all three countries, although progress is projected to be greater (1) among men than among women, and (2) among Hungarians than among Russians and Spaniards. For instance, while Hungarian men can expect to gain 13.5 additional years of life up to 2050, Russian men can expect to increase their life expectancy by 13 years, and Spanish men by 10 years. Advances are expected to be only slightly smaller among women: in Hungary and Russia, women can expect to gain 11 years, while in Spain they can expect to gain nine additional years of life up to 2050. These findings also suggest that the mortality gap between women and men will narrow, because men are more likely to catch up to the lower mortality levels of women than women are to experience additional progress at even more advanced ages. This is especially the case in Hungary and Russia, the two countries in our forecast setting in which men experienced higher mortality during the social, political, and economic transformation after the dissolution of the Soviet Union. Despite the larger projected gains in life expectancy in Hungary and Russia, Spain is likely to continue to have the highest life expectancy among the selected countries: in 2050, life expectancy is projected to reach 93.84 years for women and 88.51 years for men in Spain, but only 89.34 years for women and 84.09 years for men in Hungary, and just 85.83 years for women and 75.97 years for men in Russia.

#### Forecast uncertainty.

The gradually widening prediction intervals indicate growing uncertainty over time. For instance, the life expectancy of Hungarian men will range with a probability of 95 % from 71.15 to 75.18 years in 2020 and from 73.71 to 88.57 years in 2050. Thus, the uncertainty range almost quadruples from 4 to 15 years in only 30 forecast years. Moreover, our model assigns larger degrees of uncertainty to male than to female mortality forecasts. This is in part because male life expectancy developed less regularly than female life expectancy in the base period, an effect that is particularly pronounced in Russia: while the 95 % prediction interval includes 18 years for Russian males and 13 years for Russian females in 2050, it only comprises 15 years for men and 12 years for women in Hungary, and 13 years for men and 10 years for women in Spain.

Although our model forecasts that life expectancy will increase for women and men in Spain, Hungary, and Russia, it is important to keep in mind that these results will be valid only if the underlying assumptions turn out to be accurate.

**Table 1:** Estimates from our model: Forecasted quantiles 0.025, 0.5, and 0.975 for female (“F”) and male (“M”) life expectancy at birth in Spain, Hungary, and Russia for the years 2010, 2020, 2030, 2040, and 2050

Country	Quantile	Year									
		2010		2020		2030		2040		2050	
		F	M	F	M	F	M	F	M	F	M
Spain	0.025	84.63	78.55	85.32	79.25	85.97	79.93	86.58	80.60	87.18	81.27
	0.5	84.8	78.73	87.13	81.21	89.39	83.64	91.62	86.07	93.84	88.51
	0.975	84.87	78.86	87.98	82.66	91.08	86.44	94.18	90.20	97.18	93.86
Hungary	0.025	78.31	70.29	79.16	71.15	79.98	72.01	80.78	72.86	81.56	73.71
	0.5	78.51	70.54	81.39	73.97	84.16	77.43	86.82	80.81	89.34	84.09
	0.975	78.60	70.65	82.39	75.18	86.16	79.74	89.89	84.24	93.53	88.57
Russia	0.025	74.79	62.82	75.71	63.77	76.56	64.67	77.36	65.55	78.12	66.40
	0.5	74.98	63.04	77.75	66.21	80.45	69.38	83.13	72.62	85.83	75.97
	0.975	75.11	63.25	79.26	68.55	83.33	73.88	87.37	79.21	91.34	84.47

## Summary and concluding remarks

How do economic conditions in general, and major economic changes in particular, influence mortality; and which methodological features does a model need to be able to predict the resulting mortality developments? Although many studies have investigated the impact of economic conditions on mortality levels, the direction, the intensity, and even the existence of this relationship continue to be debated. Nevertheless, major transformation processes, including those resulting from economic and political reforms, appear to have an effect on life expectancy, especially if the changes are radical and rapid, and affect people in unexpected ways in multiple areas of life. These crises are often accompanied by rising income inequality, a deterioration in the social safety net, and material hardship; which in turn lead to increasing levels of psychosocial stress, and temporary periods of stagnating and even declining life expectancy. Spain, Hungary, and Russia experienced such irregular mortality developments due to radical socioeconomic and/or political changes. For instance, life expectancy declined in Hungary and Russia in the early 1990s following the dissolution of the Soviet Union, whereas Spain experienced large survival improvements in the 1970s following a transition to a democratic regime. Today, Spain is still feeling the effects of the financial crisis of the late 2000s, which caused, inter alia, high unemployment rates and thus high levels of social and economic insecurity—issues that raise the question of whether past increases in Spanish life expectancy are likely to continue into the future.

Irregular mortality developments are particularly difficult to forecast due to major changes in long-term trends. Many mortality forecasting approaches, such as the original Lee-Carter model (1992), cannot adequately incorporate trend reversals, since the model is based on the assumption that relative mortality changes among ages and among adjacent years are time-invariant. Recently developed approaches have attempted to overcome these shortcomings. For instance, Mitchell (2013) and Haberman and Renshaw (2012) suggested forecasting the rates of mortality improvement (rather than the death rates) in order to model flexible changes in survival over age and time. Li and Lee (2005) and Cairns et al. (2011) recommended jointly forecasting the mortality of multiple (sub)populations in so-called *coherent* mortality forecasts, which allows the user to adjust the mortality trend in each country using mortality information of other countries. We adopted these new approaches in our mortality forecasting model (Bohk and Rau 2014a), which can (1) model dynamic shifts in survival improvements from younger to older ages (using the rates of mortality improvement), and (2) adjust the mortality trend in a country of interest using information from selected reference countries.

We have employed our model to forecast irregular mortality in Spain, Hungary, and Russia, both retrospectively from 1991 to 2009; and prospectively from 2010 to 2050. While the prospective results suggest that life expectancy is likely to increase in all three countries, and that the degree of uncertainty is greater for men than for women (because male mortality fluctuated more strongly in the past), the retrospective results suggest that our model can forecast irregular mortality with changing long-term trends, and that its forecast errors are usually smaller than those of other applied models, such as the original Lee-Carter model (1992) or its coherent variant (Li and Lee 2005). However, the forecasting performance of our model is limited (like that of every model): if mortality developments become extremely volatile—as was the case in Russia after the dissolution of the Soviet Union—it seems that generating a precise forecast will depend more on luck than on methodology and expert judgment. That is why all of the retrospective forecasts of Russian life expectancy differ significantly from the observed mortality developments, whereas the models generated considerably smaller forecast errors for Hungary (and Spain). This may be in part because, like Russia, Hungary experienced a multidimensional crisis after the dissolution of the Soviet Union in which mortality increased temporarily; whereas, unlike Russia, Hungary continued to move toward democracy and capitalism. Hungary has registered large increases in life expectancy since the mid-1990s, largely due to increasing investments in the health care system, which in turn led to a reduction in the numbers of premature death due to alcohol consumption, tobacco use, and accidents; and in mortality from cardiovascular disease. In Russia, these developments appear to have occurred 10 years later (Shkolnikov et al. 2013).

What conclusions can be drawn from these findings regarding the effects of economic changes on mortality and its predictability? Although we think that there is no ultimate answer to the questions raised here, we conclude that economic changes appear to have minor effects on life expectancy in industrialized countries, but that their impact increases if they occur in conjunction with other major social and political changes. Regarding predictability, we conclude that while our model can catch turning points in long-term trends more precisely than many other models, if these radical changes occur frequently within short intervals of time, they can neither be anticipated nor forecasted by an expert and/or model.
